# Effects of Fiber Diameter on Crack Resistance of Asphalt Mixtures Reinforced by Basalt Fibers Based on Digital Image Correlation Technology

**DOI:** 10.3390/ma14237426

**Published:** 2021-12-03

**Authors:** Zhaohui Pei, Keke Lou, Heyu Kong, Bangwei Wu, Xing Wu, Peng Xiao, Yanjuan Qi

**Affiliations:** 1 College of Civil Science and Engineering, Yangzhou University, Yangzhou 225100, China; mz220200335@yzu.edu.cn (Z.P.); lkkyzu@163.com (K.L.); kongheyu@163.com (H.K.); wubw@yzu.edu.cn (B.W.); mx120190452@yzu.edu.cn (X.W.); qi_yanjuan@163.com (Y.Q.); 2 Research Center for Basalt Fiber Composite Construction Materials, Yangzhou 225127, China

**Keywords:** asphalt mixture, basalt fiber, crack resistance, different diameters, digital image correlation technique

## Abstract

It is now more popular to use basalt fibers in the engineering programs to reinforce the crack resistance of asphalt mixtures. However, research concerning the impact of the basalt fiber diameter on the macro performance of AC-13 mixtures is very limited. Therefore, in this paper, basalt fibers with three diameters, including 7, 13 and 25 μm, were selected to research the influences of fiber diameter on the crack resistance of asphalt mixtures. Different types of crack tests, such as the low temperature trabecular bending test (LTTB), the indirect tensile asphalt cracking test (IDEAL-CT), and the semi-circular bend test (SCB), were conducted to reveal the crack resistance of AC-13 mixtures. The entire cracking process was recorded through the digital image correlation (DIC) technique, and the displacement cloud pictures, strain, average crack propagation rate (V) and fracture toughness (FT) indicators were used to evaluate the crack inhibition action of the fiber diameter on the mixture. The results showed that the incorporation of basalt fiber substantially improved the crack resistance, slowed down the increase of the displacement, and delayed the fracture time. Basalt fiber with a diameter of 7 μm presented the best enhancement capability on the crack resistance of the AC-13 mixture. The flexibility index (FI) of the SCB test showed a good correlation with V and FT values of DIC test results, respectively. These findings provide theoretical advice for the popularization and engineering application of basalt fibers in asphalt pavement.

## 1. Introduction

With the continuously increased traffic load and frequent extreme environments, most asphalt pavements have not reached the design service life as they have various forms of damage, such as rutting, water damage, and cracking, etc. [1,2,3]. The cracks on the pavement could induce the stress concentration phenomenon in the asphalt mixtures [4,5,6], which could accelerate the destruction of the pavement structure, and impose a severe impact on the driving experience [7,8,9].

Engineering practices and research has shown that the introduction of glass fibers, steel fibers, polyester fibers, basalt fibers, and other fibers is a feasible way to enhance crack resistance of asphalt binders or mixtures [10,11,12,13,14]. Hasan et al. [15] studied the impact of glass fibers on performance of asphalt mixture. They believed that the anti-crack property was best when the content of glass fiber accounted for 0.12% of the total weight of the mixture. Pei et al. [16] pointed out that the addition of steel fiber improved the flexural tensile strength and anti-deformation ability of asphalt mixtures. Chen et al. [17] proposed that polyester fibers could enhance the bending and tensile properties of mixtures. Wu et al. [18] focused on the experimental research on the performance of basalt fiber-modified asphalt mixture. In his research, the optimal fiber weight content was 0.3%. Basalt fiber-reinforced asphalt mixture is a typical multiphase composite material, and it mainly enhances the performance of the mixture through fiber reinforcement and crack resistance. Additionally, during a forum about the transportation engineering technology, the China Highway Society specially organized a report on the development and innovative application of a basalt fiber transportation industry. At present, many studies on basalt fiber mainly focuses on the impact of fiber length or weight content on the performance of the mixture [19,20,21]. As a matter of fact, fiber diameter is also an important parameter needed to be taken into consideration in the design of mixtures. Fiber diameter affects the modulus of the fiber, thereby affecting its adsorption, reinforcement, crack resistance, toughening, and other functions. Therefore, this paper conducted studies about effect of fiber diameter on the crack resistance of the asphalt mixture used on the pavement.

As for the testing methods of the crack resistance of asphalt mixtures, the low temperature trabecular bending test (LTTB), indirect tensile asphalt cracking test (IDEAL-CT), and semi-circular bend test (SCB) were used to study the crack modes of the mixtures [22,23,24]. Although the selected methods can effectively distinguish the crack indexes under multiple modes, it is difficult to track and describe the cracking process. Digital image correlation (DIC) technology can track the displacement and strain of the whole field, and identify the change during the whole process [25,26]. Xing et al. [27] believed that the study of local deformation was the basic study on the failure behavior of asphalt mixtures. DIC technology is used to study the local deformation of the mixture, and the critical stable strain is proposed according to the deformation characteristics. Zhang [28], through computer simulation and actual experiments, showed that DIC art could better handle illumination changes during the measurement surface of the object or gradation change, and could accurately measure the displacement. Gou et al. [29] showed that the performance of asphalt concrete depends on its crack resistance, and digital image correlation measurement provides a method to evaluate the full-field response of asphalt concrete fracture. Buttlar et al. [30] introduced the evaluation of the application of DIC in the crack evolution process of asphalt mixtures. In view of the anisotropy and heterogeneity of asphalt mixtures, DIC technology has the advantages of non-contact, versatility, robustness, and ease of use. Therefore, it can reflect the whole process of crack formation of the mixture, and accurately capture the real displacement and strain distribution on the surface of the asphalt mixture, which is a reasonable method to study the crack evolution behavior of the asphalt mixture.

Therefore, with the aim to explore the influence of basalt fiber diameter on the crack resistance of asphalt pavement, this paper studies the enhancement effect of basalt fiber with three diameters (7, 13 and 25 μm) on the anti-crack ability of mixtures. AC-13 (AC with the aggregate nominal maximum particle size of 13.2 mm) asphalt mixture containing basalt fibers with different diameters was selected for this study. At the same time, the LTTB test, IDEAL-CT test, and SCB test were adopted to reveal the crack resistance of the mixtures. In addition, digital image correlation analysis based on the SCB test is adopted to further analyze the performance of the mixtures. The real-time orthogonal displacement field of the crack tip was calculated by capturing the full-field displacement data during the crack occurrence and development process of the asphalt mixture specimen. The dynamic fracture characteristic parameters, such as average expansion speed etc., were proposed, and the damage evolution characteristics of basalt fiber asphalt mixture were analyzed in many aspects. This research not only has importance for the selection of basalt fiber in design of asphalt mixture, but also provides an important method to construct the pavement with good crack resistance.

## 2. Materials and Test Method

### 2.1. Raw Materials

#### 2.1.1. Asphalt

SBS modified asphalt (PG 76-22) was purchased from Nantong Tongsha Asphalt Technology Co., Ltd., Nantong, China. The properties of the SBS modified asphalt are shown in Table 1. Details about the experiment methods are available in [31].

#### 2.1.2. Fiber

**Figure 1:**
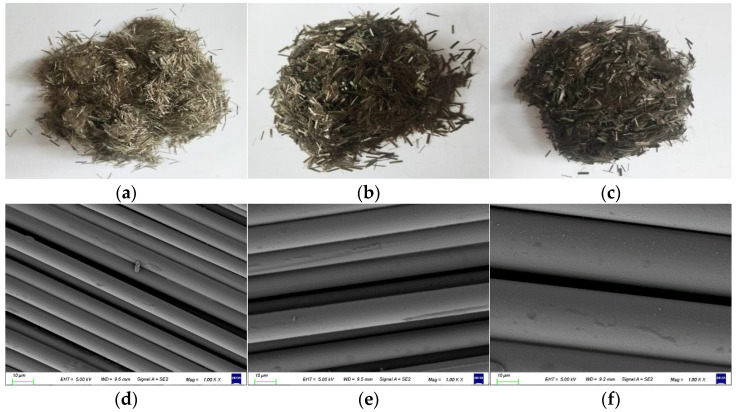
Macroscopic and microscopic morphologies of basalt fiber: (**a**) 7 μm (macro picture); (**b**) 13 μm (macro picture); (**c**) 25 μm (macro picture); (**d**) 7 μm (micro picture); (**e**) 13 μm (micro picture); (**f**) 25 μm (micro picture).

In this paper, basalt fiber adopted was purchased from Jiangsu Tianlong Basalt Continuous Fiber Co., Ltd., Yangzhou, China. Fiber is golden brown in appearance, and hard in texture. According to industrial production process standards, three basalt fibers with diameters of 7 μm, 13 μm and 25 μm are selected (the length is 6 mm), and their performance indexes are shown in Table 2. The macroscopic and microscopic morphologies of the three fibers with different diameters are shown in Figure 1.

### 2.2. Gradation Design of Asphalt Mixture

**Figure 2:**
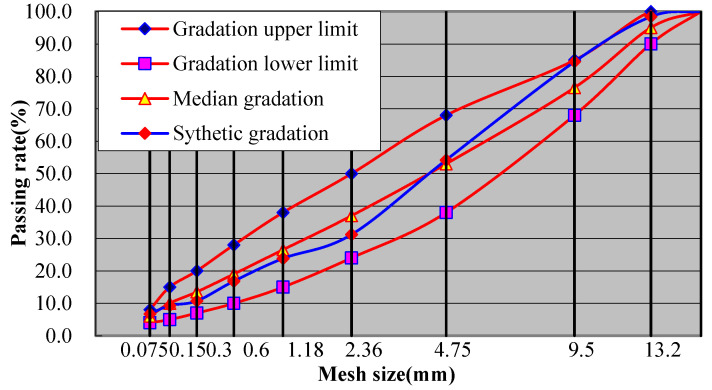
AC-13 design grading curve.

The design of the AC-13 asphalt mixture is carried out based on the specification JTG F40-2004 [32]. The grading curve of the mixture is shown in Figure 2.

Preliminary research carried out by our research group and actual engineering experience [33] suggested the length and weight content of basalt fiber as 6 mm and 0.3%, respectively. Through the Marshall test, the optimal asphalt content (OAC) is decided in the research. The gradation design results of the basalt fiber reinforced AC-13 grade asphalt mixture are listed in Table 3. Table 3 showed that OAC is higher with a smaller fiber diameter. This is because the SBS modified asphalt is a thermoplastic elastomer with good flexibility and deformability, and the fibers could absorb more asphalt in the mixture. Therefore, the fibers and asphalt together play the role of toughening the mixture.

### 2.3. Test Method

#### 2.3.1. Low Temperature Trabecular Bending Test

**Figure 3:**
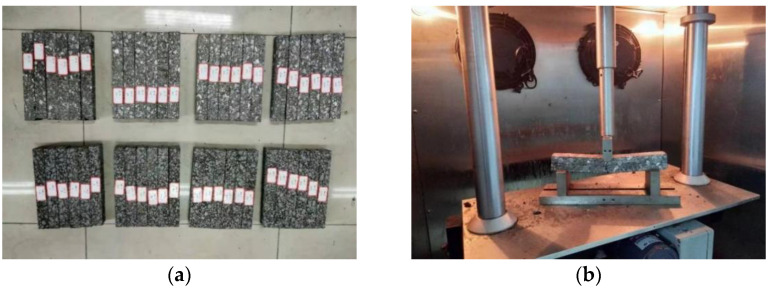
Low temperature trabecular bending test: (**a**) forming trabecular specimen; (**b**) test photo.

The low temperature trabecular bending test is a modern test method adopted by the Chinese Specification of JTG E20-2011 [31]. This test was performed on four kinds of asphalt mixtures to evaluate the crack resistance of different basalt fiber modified asphalt mixtures at low temperatures. First, the wheel-rolling method was used to form the rut plate test sample with a size of 300 mm × 300 mm × 50 mm. Then, it was cut into trabecular specimens for use, the specimen being 250 mm × 30 mm × 35 mm. The test temperature was −10 °C during the test, and the loading speed was 50 mm/min. Each group of tests had six parallel specimens, and the test specimens and process are shown in Figure 3a,b. In order to obtain more reliable values, the experiments involved in this paper were carried out three times, and the average value of data was used as the final result.

The equations used to calculate the flexural tensile strength (*R_B_*), the maximum flexural tensile strain (*ε_B_*) at the bottom of the beam, and the flexural stiffness modulus (*S_B_*) are shown in Equations (1)–(3):

(1) $$R_{B}=\frac{3LP_{B}}{2bh^{2}}$$

(2) $$\varepsilon_{B}=\frac{6hd}{L^{2}}$$

(3) $$S_{B}=\frac{R_{B}}{\varepsilon_{B}}$$

where

*b =* the width across the interruption surface (mm); 

*h* = the height across the discontinuity (mm); 

*L* = specimen length (mm); 

*P_B_* = the maximum load when the specimen breaks (N); 

*d* = mid-span displacement when the specimen fails (mm).

#### 2.3.2. IDEAL Cracking Test

**Figure 4:**
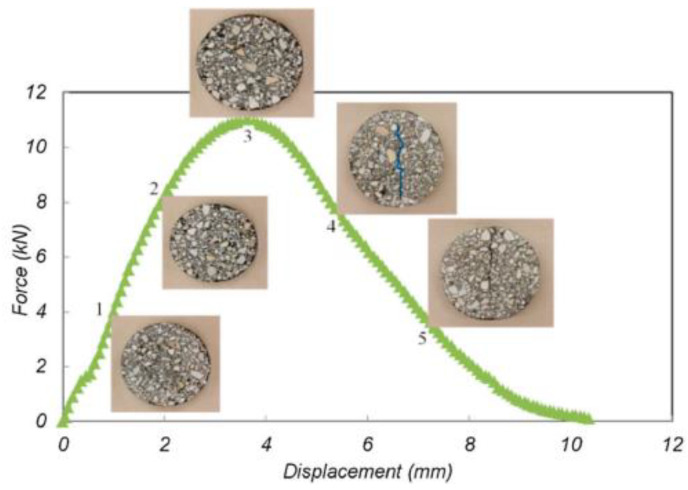
IDEAL cracking test load-displacement curve.

The IDEAL-CT test was developed by Texas Transportation Institution. This test method performs an IDEAL-CT test, which is conducted on the mixture according to the test procedure, and divides the cracking process of the asphalt mixture into three stages, which are shown in Figure 4 [34]: The first stage is the crack initiation stage, from 0 points to 3 points: the specimen cracks do not appear. The second stage is the crack longitudinal expansion stage, that is, from 3 points to the end: the cracks continue to develop until the specimen is destroyed. The third stage represents the expansion of lateral cracks and runs through the second stage.

**Figure 5:**
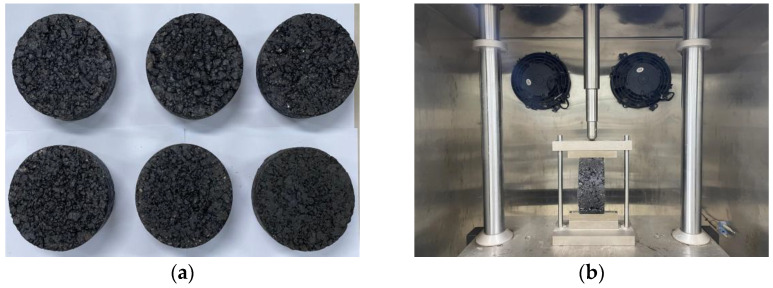
IDEAL cracking test: (**a**) forming trabecular specimen; (**b**) test photo.

This test used a cylindrical specimen formed by the rotary compactor. The dimensions of the specimen were generally 150 mm in diameter and 62 mm in thickness. The whole test was conducted at 25 °C, and the loading speed of the loader was 50 mm/min, and the loading-deformation curve was recorded. The test specimen and process are illustrated in Figure 5a,b. The cracking test indexes are listed in Equations (4) and (5).

(4) $${\mathrm{CT}}_{\mathrm{index}}=\frac{G_{f}}{\left|m_{75}\right|}\times (\frac{l_{75}}{D})$$

(5) $$\left|m_{75}\right|=\left|\left(p_{85}-p_{65}\right)/\left(l_{85}-l_{65}\right)\right|$$

where 

CT_index_ = the cracking test index; 

*G_f_* = the fracture energy (J/m^2^); 

|*m*_75_| = the slope at 75% peak after the peak point of the test curve; 

*l*_75_ = the displacement at 75% peak after the peak point of the test curve (mm); 

*D =* the diameter of the specimen (mm).

#### 2.3.3. SCB Test

**Figure 6:**
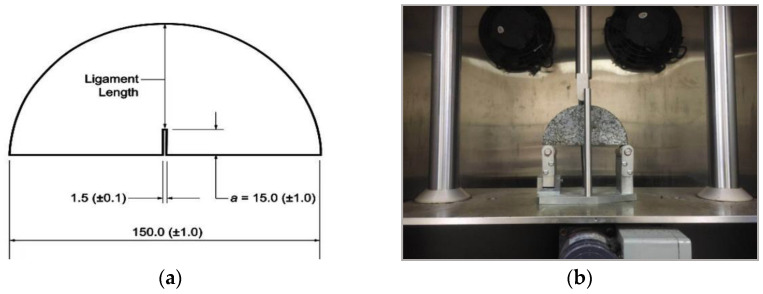
SCB test: (**a**) schematic diagram of pre-cut seam length; (**b**) test photo.

The SCB test is illustrated in the Specification of AASHTO TP 105-13 [35]. The dimensions of the semi-cylindrical specimen were 150 mm in diameter and 50 mm in thickness. The test specimen needs to reserve a certain length of reserved slit at the bottom of the test piece in advance, as shown in Figure 6a, to reveal the crack resistance of mixtures with initial cracks, and the expansion of the crack after it occurs.

**Figure 7:**
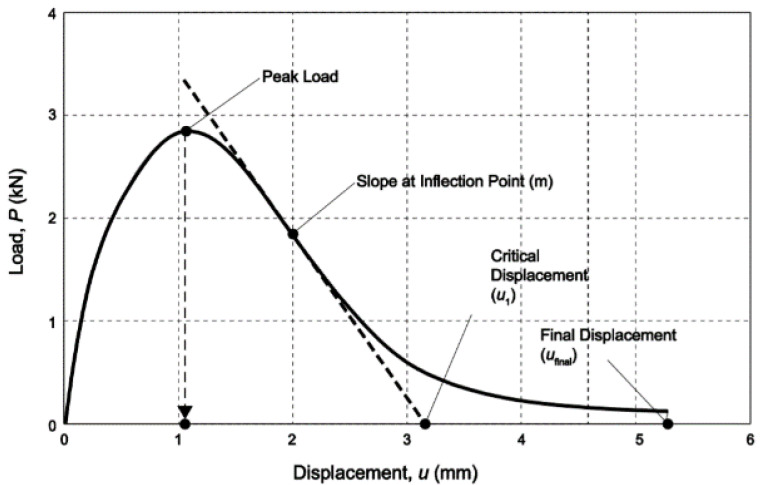
SCB test load-displacement curve.

For the SCB test, a semi-cylindrical shaped mixture specimen was placed in universal testing machine, and then the load was added by the upper metal column (Figure 6b). The test was conducted at 25 °C, while the loading speed was 50 mm/min, and the loading-deformation curve was recorded (Figure 7). The flexibility index can be expressed by Equations (6) and (7).

(6) $$G_{f}=\frac{W_{f}}{{\mathrm{Area}}_{\mathrm{lig}}}\times 10^{6}$$

(7) $$\mathrm{FI}=\frac{G_{f}}{\left|m\right|}\times A$$

where 

*G*_f_ = the fracture energy (J/m^2^); 

W_f_ = the work of fracture (J); 

Area_lig_ = the toughness area (mm^2^); 

FI = Flexibility index; 

|m| = the absolute value of the slope at the inflection point (KN/mm); 

A = the unit conversion and scaling constant (equal to 0.01).

#### 2.3.4. DIC Method Introduction

Digital image correlation technology is a test method that combines modern image programming technology with optical measurement analysis. The image displacement data of the semicircular specimen surface at different fatigue loading periods can be collected through the digital camera pixels (Sony Corporation, Shanghai, China). The basic principle of DIC is to collect the region of interest (ROI) in the pre-deformed image as a reference image, and perform network division. The target image sub-area with the best correlation with the reference image sub-area is searched for in the deformed target image through a correlation search algorithm. Then, the displacement of the sub-area is calculated, to obtain the actual displacement of the point to be measured by conversion. Finally, the Cauchy equation is used to convert the displacement data into strain data.

**Figure 8:**
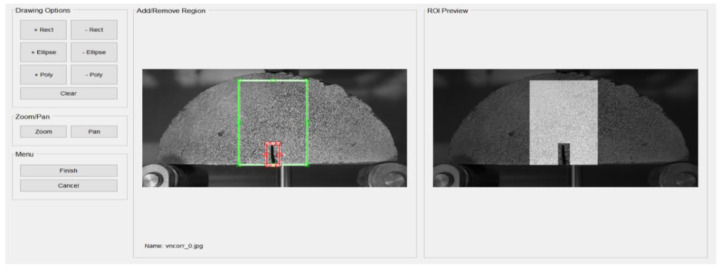
Set the ROI of the image.

**Figure 9:**
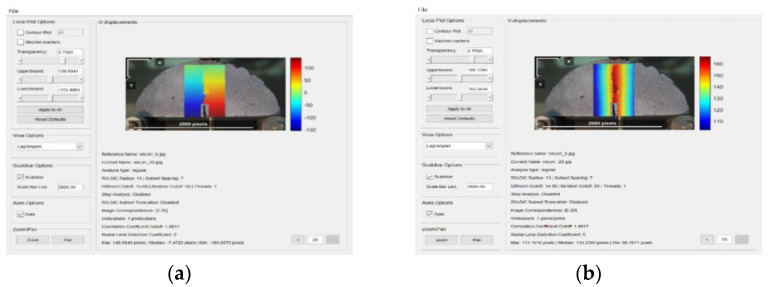
Calculation of specimen displacement: (**a**) horizontal displacement; (**b**) vertical displacement.

**Figure 10:**
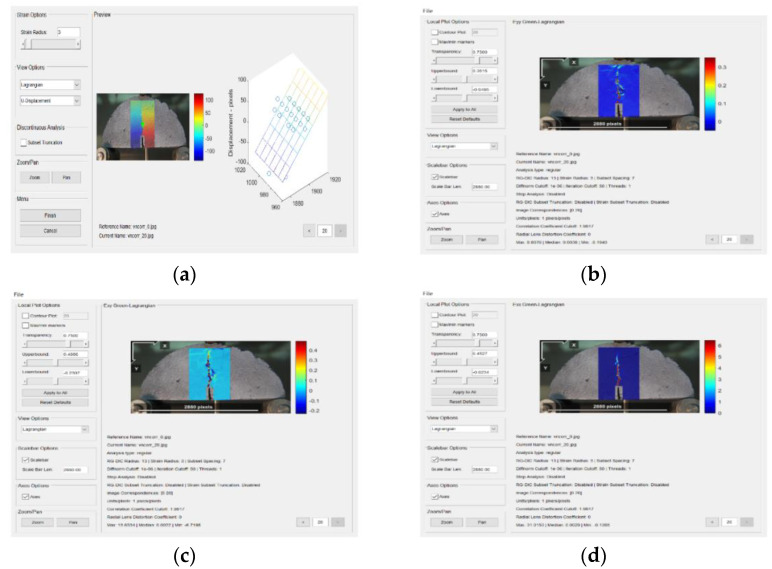
(**a**) Set a reasonable strain radius; (**b**) strain E_xx_; (**c**) strain E_yy_; (**d**) strain E_xy_.

DIC analysis was conducted using the Ncorr program in the Matlab software (Matlab 2019a). First, the reference image and the current image are inputted, and the ROI of the image is set (Figure 8). Then, the DIC parameters are set, which include subset options, iterative solver options, multi-threaded options, high strain analysis, discontinuous analysis, etc., and the position of the nucleus is set. After the nucleus is implanted, the study area is divided, each area is calculated separately, and the correlation and convergence of the reference image are calculated iteratively to increase the accuracy. The horizontal and vertical displacement of the specimen are then calculated (Figure 9). Finally, by setting a reasonable strain radius (Figure 10a) [36], after the calculation is completed, strain calculation results of the specimen can be viewed (Figure 10b–d): Strain E_xx_ (Positive strain of *x*-axis direction), strain E_yy_ (Positive strain of *y*-axis direction) and strain E_xy_ (Shear strain perpendicular to *x*-axis in *y*-axis direction) [29].

Only from the displacement and strain pictures can the fracture characteristics of asphalt mixtures be evaluated qualitatively. With the aim of quantifying the dynamic crack formation characteristics of asphalt mixtures modified by basalt fiber with different diameters, indicators, such as the real-time crack propagation length (RCPL), the average crack propagation rate (V) and the fracture toughness (FT), are introduced.

The real-time crack propagation length during the crack propagation process on the surface of the composite material is calculated by the Equation (8):

(8) $$L=\sum_{i=1}^{n}\sqrt{{\left(x_{i+1}-x_{i}\right)}^{2}+{\left(y_{i+1}-y_{i}\right)}^{2}}\times \delta$$

where

*L* = Crack length (mm); 

*δ* = Pixel conversion factor for the size of the photo.

The average crack propagation rate on the surface of the composite material is calculated by the Equation (9):

(9) $$V=\frac{L_{16}}{t_{16}}$$

The fracture toughness is calculated by the Equation (10). It is the case where there are cracks or crack-like defects in the test piece. The resistance value was displayed by the material when the so-called unstable fracture occurs:

(10) $$K=\sigma \sqrt{\pi a}$$

where 

*K* = Fracture toughness (MPa·m^1/2^); 

σ = stress (Mpa); 

a = Semicircle size factor.

## 3. Results and Discussion

### 3.1. Low Temperature Trabecular Bending Test

**Figure 11:**
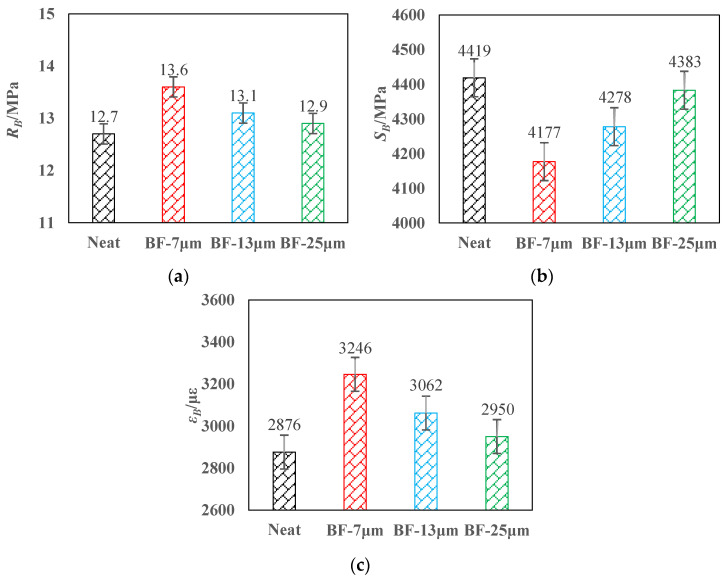
Low temperature trabecular bending test results: (**a**) flexural tensile strength; (**b**) flexural stiffness modulus; (**c**) maximum flexural tensile strain.

The LTTB test results are shown in Figure 11. Compared with the mixture without basalt fibers, the flexural strength of AC-13 modified 7, 13, and 25 μm basalt fiber increased by 7.1%, 3.1%, and 1.6%, respectively and the maximum flexural tensile strain increased by 12.8%, 6.4%, and 2.6%, respectively. Meanwhile, the flexural stiffness modulus of AC-13 decreased by 5.5%, 3.1%, and 0.8%, respectively.

Generally, the low temperature performance of three kinds of basalt fiber reinforced asphalt mixtures is slightly increased, while the flexural stiffness modulus is decreased. The explanation may be that the asphalt hardens and becomes brittle under low temperature conditions, which makes the adhesion performance between the fiber and the asphalt poor, resulting in an insignificant bridging effect [37].

### 3.2. IDEAL Cracking Test

**Figure 12:**
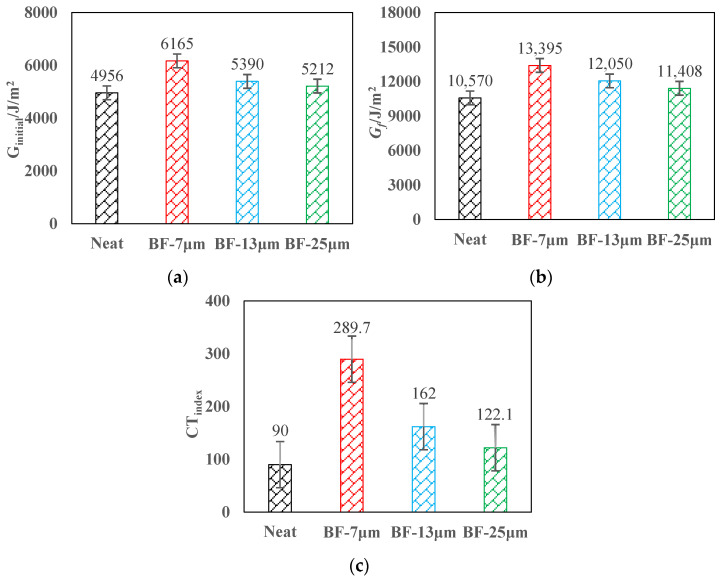
IDEAL cracking test results: (**a**) initiation energy; (**b**) fracture energy; (**c**) cracking test indexes.

Figure 12 illustrates the test results. Compared with the AC-13 mixture without basalt fiber, the initiation energy, fracture energy, and cracking test indices of the specimens having basalt fiber increased substantially. The initiation energy of AC-13 with fiber diameters of 7, 13, and 25 μm increased by 24.4%, 8.8%, and 5.2%, respectively, the fracture energy increased by 26.7%, 13.4%, and 7.9%, respectively, and the CTindex increased by 221.8%, 80%, and 35.7%, respectively.

These data indicate that basalt fiber can enhance the crack flexibility of the AC-13 mixture, which plays a role in toughening. The explanation may be that the fibers overlap with each other in the asphalt mixture, and bonds with the asphalt to create a toughening effect, which can dissipate a part of the stress concentration. At the same dosage, the finer the fiber, the larger the OAC and the more asphalt content, the better the crack resistance of the mixture. Therefore, basalt fiber with a diameter of 7 μm can best improve the crack resistance of AC-13, followed by the 13 and 25 μm basalt fiber.

### 3.3. SCB Test

**Figure 13:**
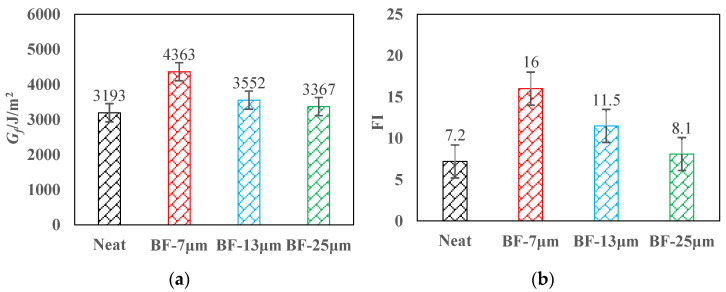
SCB test results: (**a**) fracture energy; (**b**) flexibility index.

Figure 13 revealed that compared with the mixture without fiber, the fracture energy of AC-13 with 7, 13, and 25 μm basalt fiber increased by 36.6%, 11.2%, and 5.4%, respectively, and the flexibility index increased by 122.2%, 59.7%, and 12.5%, respectively.

The change rule of the flexibility index is consistent with that of fracture energy. This shows that basalt fibers with different diameters can greatly delay the crack development speed of the AC-13 asphalt mixture after cracking. The explanation could be that the relative displacement between the aggregates after the mixture is cracked, and the chopped basalt fibers play a reinforcing role in the mixture. This offsets a part of the stress, slows down the development rate of the cracks of the mixture, and effectively delays the propagation of cracks. At the same dosage, the thinner the fiber, the greater its roots will be, and the bridging effect is more obvious. The basalt fiber with a diameter of 7 μm greatly improves the crack resistance of the asphalt mixture, and can greatly delay the propagation rate of cracks. However, the basalt fibers with diameters of 13 μm and 25 μm have little difference in the improvement of their crack resistance. This is the same as the result of the IDEAL cracking test.

### 3.4. DIC Analysis

#### 3.4.1. Full-Field Displacement

**Figure 14:**
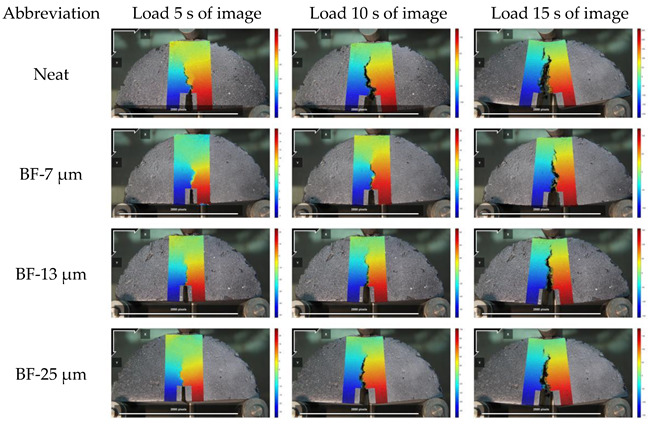
Horizontal displacement cloud pictures.

**Figure 15:**
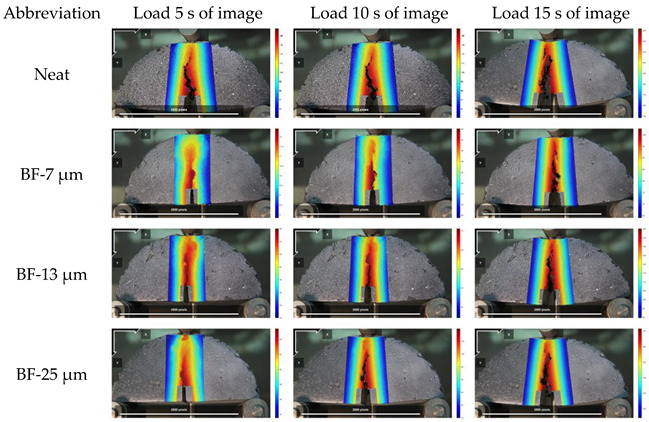
Vertical displacement cloud pictures.

The displacement diagram of the basalt fiber asphalt mixture specimens with different diameters is shown in Figure 14 and Figure 15. In the analysis, U and V are the horizontal and vertical displacement respectively.

When loading to 5 s, the displacement of the area near the crack of the semicircular specimen is small. A dark area with large displacement is created at the pre-cut, this is caused by the stress concentration generated at the notch of the specimen. As time continues to increase, the displacement cloud image shows the changing law of the bending of the semicircular specimen. When loading to 10 s, the displacement law is gradually obvious, indicating that the aggregate and asphalt mortar in the mixture begins to change synergistically. When loading to 15 s, the displacement of the mixture with and without fiber will change to different degrees. It shows that the chopped basalt fiber plays a reinforcing role in the mixture, which offsets a part of the stress and effectively slows down the overall displacement.

#### 3.4.2. Full-Field Strain

**Figure 16:**
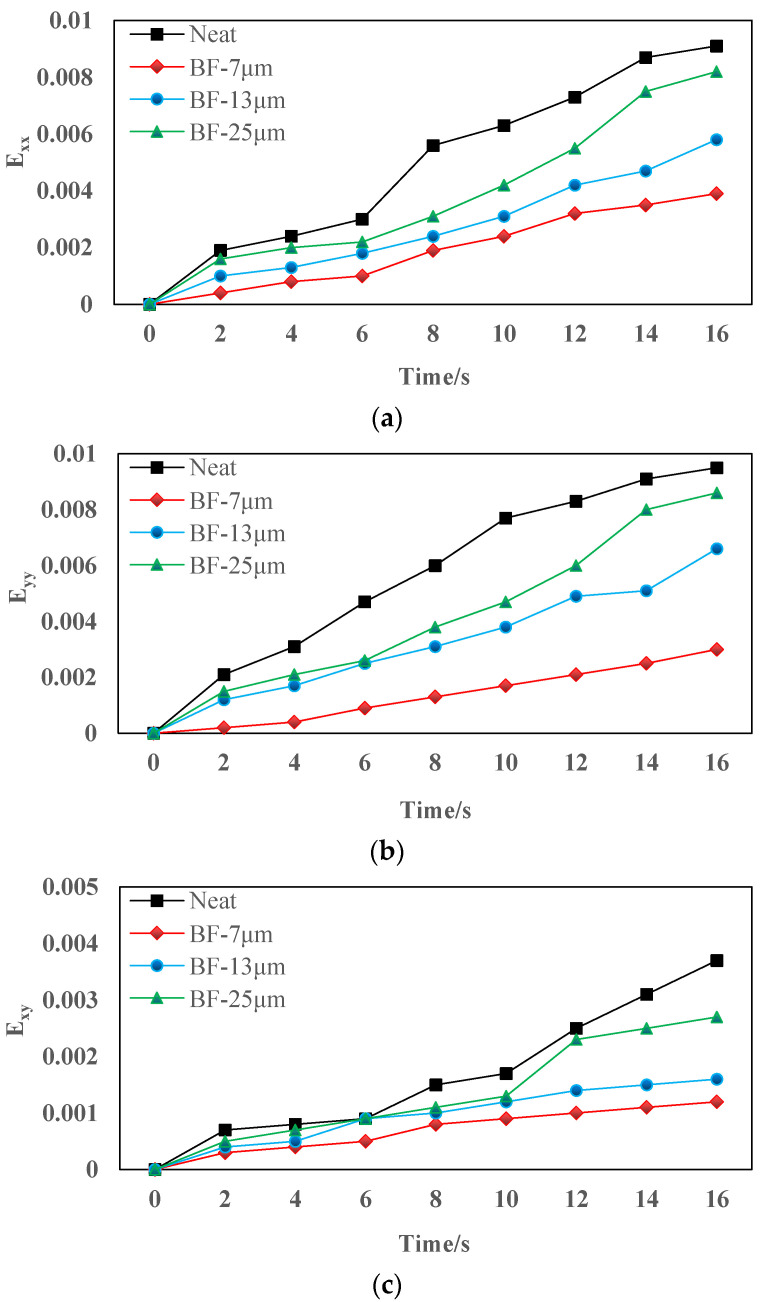
Strain test results of the specimen: (**a**) E_xx_; (**b**) E_yy_; (**c**) E_xy_.

The strain of basalt fiber reinforced mixtures with different diameters are illustrated in Figure 16. E_xx_ represents the positive strain of *x*-axis, E_yy_ represents the positive strain of *y*-axis, and E_xy_ represents the shear strain perpendicular to *x*-axis in *y*-axis direction.

Figure 16 revealed that the median strain is almost zero at the beginning, then slowly increases, and finally, increases rapidly. Therefore, the cracking process of asphalt mixture can be summarized as the crack initiation stage, the slow growth stage, and the rapid expansion stage. In the crack initiation stage, AC-13 having basalt fiber with a diameter of 7 μm significantly delayed the crack initiation time of the asphalt mixture by approximately 4 s. The basalt fibers with diameters of 13 μm and 25 μm also delayed the occurrence of cracking by about 2 s and 1 s, respectively, but the effect is not as great as the basalt fibers with a diameter of 7 μm. After adding basalt fiber, the strain in the three directions of the asphalt mixture is reduced, and the smaller the diameter of the basalt fiber, the smaller the strain of the corresponding asphalt mixture. In the AC-13 mixture, the strains E_xx_, E_xy_, and E_yy_ in the three directions all show the following trends: AC – 13 + 7 μm BF < AC – 13 + 13 μm BF < AC – 13 + 25 μm BF < AC – 13 + NO BF. When the strain is smaller, the crack resistance of the basalt fiber reinforced mixture is better. This is because, in the AC-13 specimen, the basalt fiber with a diameter of 7 μm has more specific surface area [38,39], and at the same time its number is the largest, and the total contact area with asphalt is the largest. It not only greatly improves the adhesion of the asphalt mortar, but also greatly dissipates the concentrated stress, thereby delaying the speed of crack propagation. This also complies with the test results of the macro-performance tests.

#### 3.4.3. Fracture Feature Analysis

**Figure 17:**
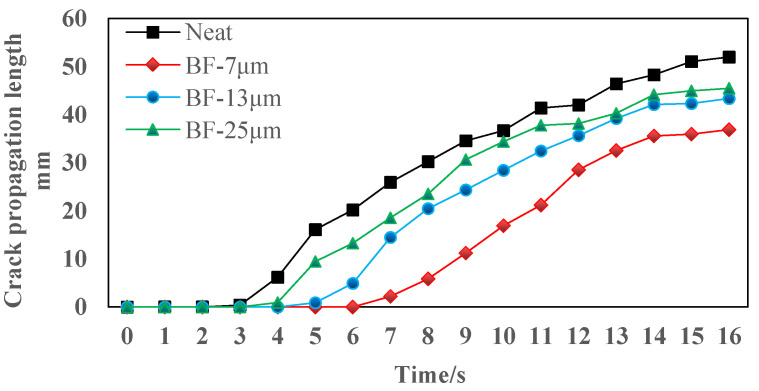
Crack propagation length of the specimen.

The real-time crack propagation length data of four kinds of asphalt mixtures are illustrated in Figure 17. The crack initiation time of BF AC-13 mixtures are longer than that of the neat mixture. This result proved that basalt fiber could enhance the crack resistance in the crack initiation stage. In addition, during the entire test, the real-time crack propagation speed of the specimen containing 7 μm basalt fiber was the smallest.

The average crack propagation rate (V) and fracture toughness (FT) are shown in Table 4. Generally, basalt fiber can increase the fracture toughness and reduce its crack propagation rate, so as to achieve the effect of toughening and cracking resistance. Compared with AC-13 without basalt fiber, fracture toughness of AC-13 asphalt mixture mixed with 7 μm BF has an increase of 27% and a decrease of 56% in the average expansion rate. This may be because the fibers can absorb part of the asphalt, thereby playing the role of reinforcement, buffering and adsorption, and improving the crack resistance. At the same time, under the same dosage, a smaller fiber diameter could result in larger surface area. Therefore, the total contact area with asphalt is bigger, which made the basalt fiber with a diameter of 7 μm present the best enhancement capability on the crack resistance of the mixture.

### 3.5. Index Correlation Analysis

**Figure 18:**
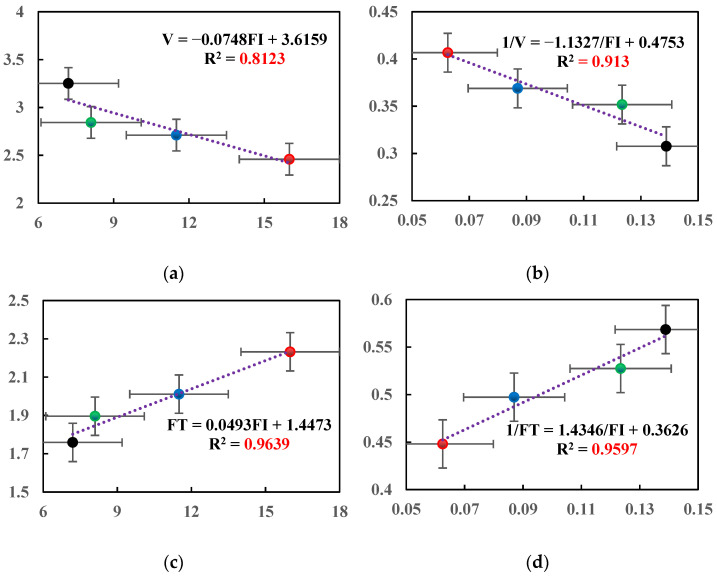
Index correlation analysis: (**a**) linear fitting of FI and V; (**b**) linear fitting of 1/FI and 1/V; (**c**) linear fitting of FI and FT; (**d**) linear fitting of 1/FI and 1/FT.

The ANOVA Tukey’s HSD analysis of indicators was carried out based on the conclusions obtained from the above experiments [39]. In order to ensure the uniformity of the sample conditions, the FI index values (based on the index obtained by the SCB test) of the four kinds of asphalt mixtures were selected to analyze the average crack propagation rate and fracture toughness, respectively. Figure 18 showed that the correlation coefficient R^2^ of the three fitting curves is above 0.9, indicating that 1/FI has a good correlation with 1/V and 1/FT, respectively. On the one hand, it is verified that the DIC technology is reasonable for evaluating the anti-damage and cracking characteristics of asphalt mixtures. On the other hand, it also shows that the relevant macroscopic test results have a good correlation with the DIC parameters.

## 4. Conclusions

In this paper, basalt fibers with diameters of 7, 13, and 25 μm were selected to study the influences of fiber diameter on crack resistance of AC-13 mixtures. Different kinds of crack tests were conducted to test the physical properties of the mixtures, and DIC technique was utilized to capture the picture of asphalt specimen in the whole process during the SCB test. The correlation analysis between FI and V as well as FT was also conducted. The conclusions drawn from the above analysis are as follows:

- Basalt fiber can improve the crack resistance of the AC-13 asphalt mixture. Among them, basalt fiber with a diameter of 7 μm has the most prominent enhancement in the crack resistance, while the reinforcing effect of basalt fibers with diameters of 13 μm and 25 μm is not much different, and both are inferior to 7 μm fibers;
- Under the same dosage, the basalt fiber with a diameter of 7 μm has the best enhancement effect on the crack resistance of AC-13 mixture;
- Basalt fiber asphalt mixture has slower crack propagation rate than asphalt mixture without fiber;
- The 1/FI of the SCB test shows a better correlation with the 1/V and 1/FT values of DIC test results, respectively.

The research only focused on extensive laboratory tests on the crack resistance of basalt fiber modified asphalt mixtures with different diameters. In future research, multiple characterization methods can be used for comprehensive analysis about of the relationship between the fiber diameter and the cracking mechanism of asphalt mixture.

## Figures and Tables

**Table 1:** Properties of SBS modified asphalt.

Test Indexes		Requirements	Results	Experiment Method
Penetration (25 °C)/0.1 mm		60~80	71	T0604
Softening point/°C		≮55	86	T0606
Ductility (5 cm/min, 15 °C)/cm		≮30	48	T0605
Penetration Index (PI)		−0.4~1.0	0.5	T0604
Segregation (soften the spread)/°C		≯2.5	1.4	T0661
Recovery of elasticity (25 °C)/%		≮65	76	T0662
RTFOT residue	Quality change/%	±1.0	−0.08	T0610
	Penetration ratio/%	≮60	86	T0604
	15 °C residual ductility/cm	≮20	37	T0605

**Table 2:** The property of basalt fiber.

Diameter/μm	Fracture Strength/MPa	Water Content/%
7	3200	0.1
13	2218	0.1
25	1940	0.1

**Table 3:** AC-13 asphalt mixture component material.

Numbering	Gradation Type	Fiber Stabilizer	Dosage/%	OAC/%	Abbreviation
1	AC-13	/	/	4.8	AC-13
2	AC-13	BF (6 mm, 7 μm)	0.3	5.1	AC-13 BF-7 μm
3	AC-13	BF (6 mm, 13 μm)	0.3	5.0	AC-13 BF-13 μm
4	AC-13	BF (6 mm, 25 μm)	0.3	4.9	AC-13 BF-25 μm

**Table 4:** Average crack propagation rate and fracture toughness test results.

Code	Neat	BF-7 μm	BF-13 μm	BF-25 μm
V (mm/s)	3.251	2.459	2.711	2.843
FT (MPa·m)	1.759	2.232	2.011	1.896

## Data Availability

Not applicable.
